# The Influence of Calcium toward Order/Disorder Conformation of Repeat-in-Toxin (RTX) Structure of Family I.3 Lipase from *Pseudomonas fluorescens* AMS8

**DOI:** 10.3390/toxins12090579

**Published:** 2020-09-09

**Authors:** Nur Shidaa Mohd Ali, Abu Bakar Salleh, Thean Chor Leow, Raja Noor Zaliha Raja Abd Rahman, Mohd Shukuri Mohamad Ali

**Affiliations:** 1 Enzyme and Microbial Technology Research Center, Faculty of Biotechnology and Biomolecular Sciences, Universiti Putra Malaysia, Serdang 43400, Malaysia; nur_shidaa@yahoo.com (N.S.M.A.); abubakar@upm.edu.my (A.B.S.); adamleow@upm.edu.my (T.C.L.); rnzaliha@upm.edu.my (R.N.Z.R.A.R.); 2 Department of Biochemistry, Faculty of Biotechnology and Biomolecular Sciences, Universiti Putra Malaysia, Serdang 43400, Malaysia; 3 Department of Cell and Molecular Biology, Faculty of Biotechnology and Biomolecular Sciences, Universiti Putra Malaysia, Serdang 43400, Malaysia; 4 Department of Microbiology, Faculty of Biotechnology and Biomolecular Sciences, Universiti Putra Malaysia, Serdang 43400, Malaysia

**Keywords:** repeat-in-toxin, lipase, metal binding protein, order/disorder, calcium ion, structure, function, folding

## Abstract

Calcium-binding plays a decisive role in the folding and stabilization of many RTX proteins, especially for the RTX domain. Although many studies have been conducted to prove the contribution of Ca^2+^ ion toward the folding and stabilization of RTX proteins, its functional dynamics and conformational structural changes remain elusive. Here, molecular docking and molecular dynamics (MD) simulations were performed to analyze the contribution of Ca^2+^ ion toward the folding and stabilization of the RTX lipase (AMS8 lipase) structure. AMS8 lipase contains six Ca^2+^ ions (Ca1–Ca6). Three Ca^2+^ ions (Ca3, Ca4, and Ca5) were bound to the RTX parallel *β*-roll motif repeat structure (RTX domain). The metal ion (Ca^2+^) docking analysis gives a high binding energy, especially for Ca4 and Ca5 which are tightly bound to the RTX domain. The function of each Ca^2+^ ion is further analyzed using the MD simulation. The removal of Ca3, Ca4, and Ca5 caused the AMS8 lipase structure to become unstable and unfolded. The results suggested that Ca3, Ca4, and Ca5 stabilized the RTX domain. In conclusion, Ca3, Ca4, and Ca5 play a crucial role in the folding and stabilization of the RTX domain, which sustain the integrity of the overall AMS8 lipase structure.

## 1. Introduction

Calcium-binding plays a decisive role in the folding and stabilization of many RTX proteins, especially for the RTX parallel *β*-roll motif repeat structure. Disorder-to-order conformation plays a crucial role in the biological function of many proteins containing intrinsically disordered domains. This trait is exhibited by the repeat-in-toxin (RTX) protein family [1]. RTX proteins represent a broad and diverse family of pore-forming protein produced by various Gram-negative bacteria. The genera *Pseudomonas* and *Serratia* produced RTX lipases of the I.3 subfamily. It contains glycine (G) and aspartate (D) nonapeptide repeats of the consensus sequence GGXGXDXUX (where X can be any amino acid and U represents hydrophobic residue) and involved with the binding of Ca^2+^ ions.

Calcium-binding plays a decisive role in the folding and stabilization of many RTX proteins, especially for the folding of the RTX parallel *β*-roll motif repeat structure (RTX domain). The Ca^2+^ ions bound to the RTX nonapeptide repeats sequence form an RTX parallel *β*-roll motif repeat structure. This calcium-binding triggers a strong reduction in the mean net charge, dehydration and compaction, folding and stabilization of secondary and tertiary structures of the RTX proteins [1]. The RTX proteins show signs of an intrinsically disordered protein in the absence of Ca^2+^ ion. Many experimental works have been conducted to prove the contribution of Ca^2+^ ion [2,3,4,5,6,7,8]. However, the study is scarce to further analyze the contribution of Ca^2+^ ion toward the folding and stabilization of the RTX protein structure through in silico approaches. Computational analyses such as molecular docking and molecular dynamics (MD) simulations would provide a better understanding of protein functions compared to laboratory experiments since the structure can be visualized and analyzed.

In silico approaches use the atomic structure of proteins and molecular modeling for the structure-function analysis. The structural conformational changes can be analyzed via in silico based on a three-dimensional (3D) structure, multiple alignments of homologous sequences, and MD simulation [9]. Previously, proteins have been viewed as static entities and their function has been explained by direct structural interactions between the protein and substrate [2]. To better understand protein structural conformation changes, laboratory experiments have been performed coupled with the in silico analysis to support the findings. Both combinations of studies give a better understanding of protein stability and structural conformation changes.

Protein stability is a critical factor affecting the structure, function, and folding of the protein structure. Protein stability is the net balance of forces, which determine whether a protein structure will result in a native-folded conformation or an unfolded state [3]. Many factors affect the stability of the protein structure such as pH, temperature, various atomic/group interactions (hydrophobic, electrostatic, hydrogen bonding, van der Waals, and disulfide), and metal ion interaction (Zn^2+^, Ca^2+^) [4,5]. The metal ion binding plays pivotal roles in protein structure, function, and stability, especially for the RTX protein structure [6,7]. Theoretically, the RTX protein structure is intrinsically disordered/unfolded in the absence of Ca^2+^ ion [10,11]. Specifically, only calcium-binding induces the formation of the RTX parallel *β*-roll motif repeats structure and maintains the overall RTX protein structure-function/stability [12,13].

In 2013, the in silico approach (homology modeling) was used to predict the 3D structure of a new RTX lipase (*Pseudomonas fluorescens* strain AMS8 [accession number ADM87309]) (Figure S1). Homology modeling is the method used for predicting the 3D protein structure in the absence of crystal structure. The AMS8 lipase classified as RTX lipases belonged to the I.3 subfamily by the presence of the RTX domain at the C-terminal and by the absence of cysteine residues. The AMS8 lipase structure consists of six Ca^2+^ ions that may stabilize the RTX domain and the overall AMS8 lipase structure [13]. In 2020, the six Ca^2+^ ions were further analyzed and labeled as Ca1, Ca2, Ca3, Ca4, Ca5, and Ca6. Ca1 and Ca2 were located at the catalytic domain, while Ca3, Ca4, and Ca5 were bound at the RTX parallel *β*-roll motif repeats structure (RTX domain) in the non-catalytic domain with Ca6 [14]. Ali et al. (2020) revealed that the AMS8 lipase consists of three RTX nonapeptide repeats sequence that built up the RTX parallel *β*-roll motif repeats structure (residue 373–405) and three of the Ca^2+^ ions (Ca3, Ca4, and Ca5) were bound to the RTX parallel *β*-roll motif repeats structure [14]. The role of Ca^2+^ ions toward the activity and folding of AMS8 lipase has been investigated using various well established biophysical tools. Based on the biophysical characterization analyses, Ca^2+^ ions play crucial roles in the activity and folding of the AMS8 lipase [14]. Since the role of calcium-binding toward the activity and folding of AMS8 lipase has already been proved through laboratory experiments (biophysical characterizations), we further analyzed the function of Ca^2+^ ions toward the AMS8 lipase structure through in silico approaches.

Thus, this research aimed to further examine the influence of each Ca^2+^ ion toward the stabilization and structural conformational changes of the overall AMS8 lipase structure through molecular docking and MD simulations.

## 2. Results and Discussion

### 2.1. Molecular Docking (Metal Ion) of AMS8 Lipase Structure

**Figure 1:**
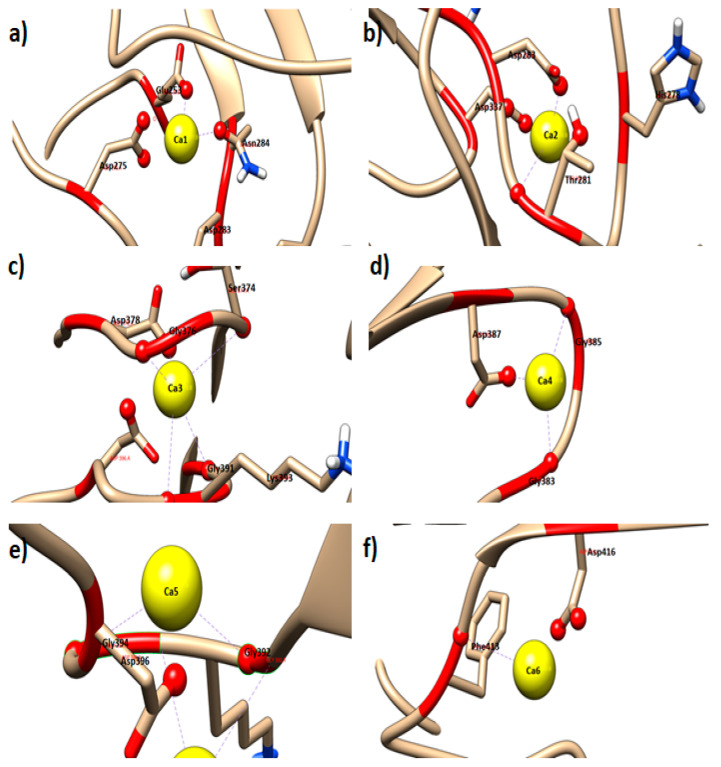
AMS8 lipase-contacting receptor residues of metal ion (Ca^2+^ ion) docking analysis. Figure 1 (**a**–**f**) represents the three-dimensional (3D) structure of AMS8 lipase-contacting receptor residues of Ca1, Ca2, Ca3, Ca4, Ca5, and Ca6, respectively.

Molecular docking was used to predict the conformation of a receptor-ligand complex. The receptor is usually a protein or a nucleic acid molecule, while the ligand can be a small molecule, another protein, or metal ion [15]. As the AMS8 lipase revealed good quality based on the verification methods of Ali et al., a docking study of Ca^2+^ ions onto the AMS8 lipase structure was performed using YASARA software (Krieger, Vienna, Austria) [16]. As a result, 25 poses were generated. After sorting the 25 runs, the following 20 distinct complex conformations were found and all differed by at least 3.0 Å heavy atom RMSd. All the poses had binding energy values in descending order from the highest to the lowest. At the end of each docking, AutoDock reports the dissociate constant (pM) and binding energy (kcal/mol). The first poses produced the highest binding energy, which further selected and analyzed (Table 1) using the Ligplot software (European Bioinformatics Institute, Cambridgeshire, UK) and visualized by using the Chimera software Resource for Biocomputing, Visualization and Informatics (RBVI), University of California, San Francisco, CA, USA) to analyze the interacting residues with the Ca^2+^ ion (Figure 1a–f).

Based on the result in Table 1, Ca1 has a binding energy of 1.288 kcal mol^−1^ with four interacting residues including Glu^253^, Asp^275,283^, and Asn^284^ (Figure 1a). Ca2 has a binding energy of 0.947 kcal mol^−1^ with four interacting residues including Asp^283,337^, His^278^, and Thr^281^ (Figure 1b). Ca3 has a binding energy of 1.084 kcal mol^−1^ with five interacting residues including Ser^374^, Asp^378^, Gly^376,391^, and Lys^393^ (Figure 1c). Ca4 has a binding energy of 1.304 kcal mol^−1^ with three interacting residues including Gly^383,385^ and Asp^387^ (Figure 1d). Ca5 has a binding energy of 1.305 kcal mol^−1^ with three interacting residues including Gly^392,394^ and Asp^396^ (Figure 1e). Ca6 has a binding energy of 0.999 kcal mol^−1^ with two interacting residues including Phe^413^ and Asp^416^ (Figure 1f).

The binding energy value of all six Ca^2+^ ions was determined as positive binding energy values. The more positive energies showed stronger binding, whereas negative energies indicated no binding [17]. Out of the six Ca^2+^ ions binding in the AMS8 lipase structure, Ca5, Ca4, and Ca1 had the highest binding energy value that was 1.305, 1.304, and 1.298 kcal mol^−1^, respectively. The Ca5 and Ca4 were found firmly bound to the RTX parallel *β*-roll motif repeat structure at the non-catalytic domain. The results suggested that Ca5 and Ca4 play a crucial role in the formation and folding of the RTX parallel *β*-roll motif repeat structure. Based on the interacting residue from the docking analysis, all Ca^2+^ ions (Ca3, Ca4, and Ca5) bound to the RTX parallel *β*-roll motif repeat structure interacted with Gly and Asp amino acid. This condition supports the theory of the formation of the RTX parallel *β*-roll motif repeat structure. Theoretically, the RTX nonapeptide repeats sequence is a Gly and Asp-rich nonapeptide that constitutes a specific calcium-binding motif. In the presence of Ca^2+^ ion, several tandemly repeated RTX nonapeptide repeats sequences can fold into a parallel *β*-roll motif repeat that consists of a succession of turns (encoded by the first six amino acids, GGXGXD, of each RTX *β*-roll motif repeat) and short *β*-strands (encoded by the three residues, XUX, of the RTX *β*-roll motif repeat) [18,19].

Previous laboratory experiments have also been conducted to prove and support that the Ca^2+^ ion was tightly bound to the AMS8 lipase structure. Biophysical characterizations show that the Ca^2+^ ion was tightly bound to the AMS8 lipase structure. The results based on the k_D_ value were analyzed by isothermal titration calorimetry (ITC). Based on the ITC analysis, we focused on the binding affinity between the protein and ligand. The binding affinity (dissociation constant [k_D_]) was used to measure the strengths of biomolecular interactions. The k_D_ value for AMS8 lipase with the ligand was 1.458 × 10^−7^ M. Since the k_D_ value observed was small, this proves that the Ca^2+^ ion was tightly bound to the AMS8 lipase. The lower the value of the k_D_, the higher the ligand’s binding affinity to AMS8 lipase. If the k_D_ value was high, the ligand would be weakly bound to the protein (other target molecules) [14]. Even though the unit measurement was used in the laboratory experiment (ITC) and the in silico study (molecular docking) was different due to the different modes of analysis, both analyses gave the same target, which focused on the binding affinity between the ligand (Ca^2+^) and protein structure (AMS8 lipase).

Based on both laboratory and docking analysis, we can see that the Ca^2+^ ion was tightly bound to the AMS8 lipase structure. It appeared to promote folding and formation of the RTX parallel *β*-roll motif repeat structure. Ca3, Ca4, and Ca5 imposed the adoption of a functional conformation on the secreted RTX proteins in the extracellular environment [8,15,17].

### 2.2. Molecular Dynamics (MD) Simulation of AMS8 Lipase Structure

#### 2.2.1. Root Mean Square Deviation (RMSd) Values of Backbone Atoms Analysis

Each experimental or computational technique probes different temporal and spatial scales, ranging from picoseconds to milliseconds and minutes and from atomic positional fluctuations to conformational changes of large domains, respectively. To study the protein folding and structural conformation changes, MD simulation can perform starting from nanoseconds (ns) up to seconds (s) [20]. However, there is no specific time scale to study protein folding and conformational structural changes. In this study, 50 ns have been chosen to perform the MD simulation of AMS8 lipase without Ca1, Ca2, Ca3, Ca4, Ca5, and Ca6, respectively. The RMSd scores of the backbone atoms in the initial models assess the convergence of the protein structure.

**Figure 2:**
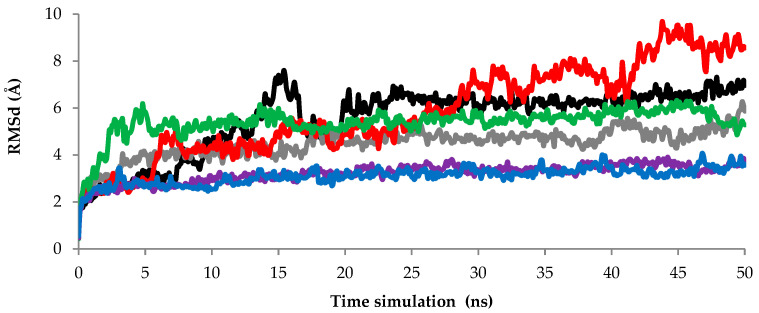
AMS8 lipase root mean square deviations (RMSd) of the backbone atoms as a function of time. The molecular dynamics (MD) simulation was conducted up to 50 ns with Ca^2+^ ions; black, grey, red, green, purple, and blue indicate the scores of RMSd without Ca1(**—**), Ca2(**—**), Ca3(**—**), Ca4(**—**), Ca5(**—**), and Ca6(**—**), respectively.

In this study, the RMSd values from the minimized predicted model structure during MD simulation with Ca^2+^ ions (Ca1–Ca6) are shown in Figure 2. The RMSd graph indicates the stability of the AMS8 lipase structure with Ca^2+^ ions. The fluctuating value of the AMS8 lipase structure without Ca1 (black line) was detected starting at 2 Å and continued to increase until 7.6 Å at 15 ns. The fluctuation started to decrease at 20 ns and maintained stable approximately 6 Å toward the end of the simulation at 50 ns. Even though the beginning of the simulation gave unstable and high fluctuations, low and stable fluctuation toward the end of the simulation indicated that the AMS8 lipase without Ca1 had become stable.

Based on the RMSd graph, the AMS8 lipase structure without Ca3 (red line) gave the highest fluctuation value from the beginning toward the end of the simulation. The RMSd values started approximately at 2 Å and continued to increase to 9.6 Å at 43 ns. These RMSd values decreased and maintained around 9 Å at the end of the simulation at 50 ns. In addition to the AMS8 lipase without Ca3, the AMS8 lipase without Ca4 (green line) also gave a high fluctuation value at the beginning of the simulation. The fluctuation value started with 6.1 Å at the beginning of the simulation at 5 ns compared to others only around 2–3 Å. However, the AMS8 lipase without the Ca4 fluctuation value maintained approximately 5 Å toward the end of the simulation. Nevertheless, the RMSd value of the AMS8 lipase predicted structure without Ca2 (grey line), Ca5 (purple line), and Ca6 (blue line) were maintained and did not deviate more than 6.0 Å toward the end of the simulation at 50 ns.

The RMSd results indicated that the AMS8 lipase structure without Ca3 looked unstable due to the highest value of fluctuation at the end of the simulation compared to others. Based on the results, only a small fluctuation has been detected until the end of the simulation, except for the removal of Ca3. This condition was due to the removal of the Ca^2+^ ion that firmly bound to the RTX domain. The binding of Ca3, Ca4, and Ca5 is involved in the RTX nonapeptide repeats sequence that will form the RTX parallel *β*-roll motif repeat structure. Based on the literature, only the Ca^2+^ ion specifically induces and stabilizes the conformation of the RTX parallel *β*-roll motif repeat structure [21]. Therefore, by removing the Ca^2+^ ion needed by the RTX nonapeptide repeats sequence to form the RTX parallel *β*-roll motif repeat structure, it makes the AMS8 lipase structure be incorrectly folded and become unstable. However, only the removal of Ca3 gives a high fluctuation until the end of the simulation. The removal of Ca^2+^ from the RTX domain caused the AMS8 lipase structure to become unstable and unfold. Since the structure already unfolds, there is no point in increasing the time simulation. Therefore, we decided to stop the simulation at 50 ns. We believed that the same pattern of graph would have been obtained if the simulation time had been added since the structure already unfolded.

#### 2.2.2. Root Mean Square Fluctuation (RMSf) Value of Residue Analysis

**Figure 3:**
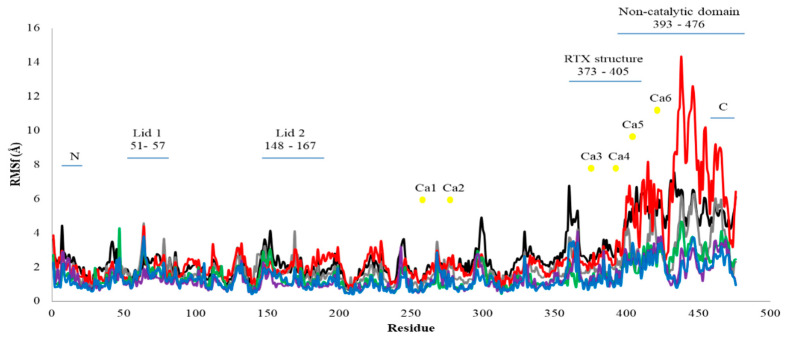
AMS8 lipase atoms root mean square fluctuations (RMSf) per residue. The MD simulation was conducted up to 50 ns with Ca^2+^ ions; black, grey, red, green, purple, and blue indicate the scores of RMSf without Ca1(**—**), Ca2(**—**), Ca3(**—**), Ca4(**—**), Ca5(**—**), and Ca6(**—**), individually.

The RMSf per residue for AMS8 lipase simulated with the presence and absence of specific Ca^2+^ ions is shown in Figure 3. The average RMSf scores per residue for all Ca^2+^ ions (Ca1–Ca6) were varied from 1.3 to 2.8 Å. At the catalytic domain, a low fluctuation was detected starting within 2–4 Å for all Ca^2+^ ions. Since the fluctuation values were low, these did not indicate higher flexibility of the residues simulated in water. The Ca1 and Ca2 showed high fluctuation values at the lid structure. Both Ca^2+^ ions located near the catalytic triad were compared to another four Ca^2+^ ions. An examination of the structural flexibility of the lid structure reveals that lid 1 had a lower average value of RMSf fluctuation (1.3 Å) compared to lid 2 (1.8 Å). Previous studies revealed that lid 2 was more flexible and it was the first lid to open when the substrates were present [22]. The high fluctuation at lid 2 suggested that the Ca^2+^ ions located in the catalytic domain (Ca1 and Ca2) might be involved in the flexibility of the lid structures.

The RMSf graph shows that higher fluctuations occurred in the non-catalytic domain. The highest fluctuation score was 14.3 Å without Ca3 (red line) at residue 438 followed by 12.5 Å without Ca3 also at residue 439. The most consistent fluctuation occurred at residues 393–476. These residues resided in the non-catalytic domain whereby the RTX parallel *β*-roll motif repeat structure was present. The flexibility of the non-catalytic domain might occur because of the presence of high Gly residues. Gly residues have introduced flexibility in the protein structure because they lack the side chain [23]. Here, AMS8 lipase also rich with Gly residue at the non-catalytic domain since the RTX nonapeptide repeats sequence consists of Gly and Asp-rich nonapeptide that constitutes a specific calcium-binding motif. The Ca3 was one of the Ca^2+^ ions that bound to the RTX parallel *β*-roll motif repeat structure. Removal of Ca3 increased the flexibility of the AMS8 lipase predicted structure.

The previous literature implied that the non-catalytic domain was a crucial part of the AMS8 lipase predicted structure. This domain contributes to the stabilization of the enzyme structure in the presence of Ca^2+^ ions [13]. In addition to the removal of Ca3, the removal of Ca1 also caused high fluctuation at the non-catalytic of the AMS8 lipase structure. Ca1 was bound to the catalytic domain and near the catalytic triad of the AMS8 lipase. In addition, Ca1 was also one of the Ca^2+^ ions that tightly bound to the AMS8 lipase structure based on molecular docking analysis. Based on the RMSf results, the fluctuation scores for the residues that interacted with Ca^2+^ ions in the catalytic domain (Ca1, Ca2, and Ca3) were maintained around 4.0 Å and not fluctuated more than 7.0 Å. Higher fluctuation scores (12.0–14.0 Å) were detected only in the non-catalytic domain. A high fluctuation value indicated that the Ca^2+^ ions contributed to the stabilization of the non-catalytic domain. High fluctuations were detected by removing Ca^2+^ ions (Ca1, Ca2, and Ca3) from the AMS8 lipase structure. Previous studies agreed that the Ca^2+^ ions played an essential role in the structural stability of the *Burkholderia glumae* lipase [24].

Experimental data on the effect of CaCl_2_ on the AMS8 lipase activity supported the idea that Ca^2+^ ions contributed to the enzyme structural stability [14]. Far-UV CD spectra revealed that the presence of CaCl_2_ improved the secondary structure of AMS8 lipase. Interestingly, the percentage of *β*-sheet (7.7–26.7%) and *α*-helix (19.4–29.7%) analyzed using CD was within the range with the AMS8 lipase structure as analyzed using the YASARA software reported by Ali et al. [13]; *β*-sheet (22.5%) and *α*-helix (27.7%). The catalytic domain was suggested to be a fundamental domain for the catalytic efficacy of AMS8 lipase [13]. The *α*/*β* structure of AMS8 was located inside the catalytic domain. Thus, this domain was proposed to be liable for the activity and stability of the protein despite the flexibility properties of the non-catalytic domain which represents a role in the structure and function of the AMS8 lipase [13]. However, the removal of Ca^2+^ ions especially, (Ca3, Ca4, and Ca5) in the non-catalytic domain caused the increment of RMSf scores, especially for the removal of Ca3 from the RTX parallel *β*-roll motif repeat structure. These indicate that destabilization and flexibility of the residues bound to Ca3 prove the critical role in stabilizing the RTX parallel *β*-roll motif repeat structure and the whole part of the non-catalytic domain.

#### 2.2.3. Solvent Accessible Surface Area (SASA) Analysis

**Figure 4:**
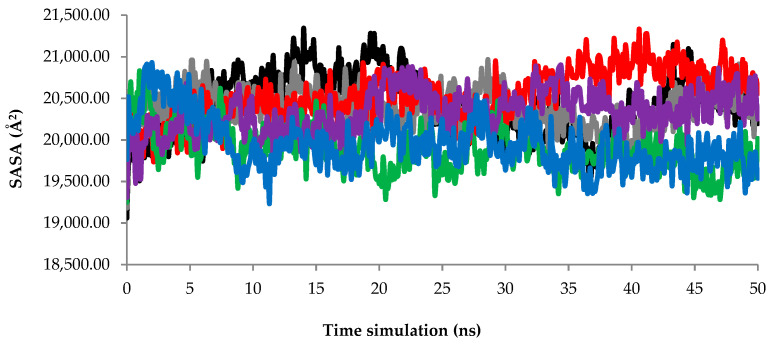
AMS8 lipase solvent accessible surface area (SASA) scores. The MD simulation was conducted up to 50 ns with Ca^2+^ ions; black, grey, red, green, purple, and blue indicate the scores of SASA without Ca1(**—**), Ca2(**—**), Ca3(**—**), Ca4(**—**), Ca5(**—**), and Ca6(**—**), individually.

The solvent accessible surface area (SASA) was also used to study the solvent-accessible surface area of AMS8 lipase. SASA indicates the transfer of free energy required to move a protein from aqueous to a non-polar solvent [25]. Based on the SASA graph in Figure 4, the AMS8 lipase structure without the Ca2, Ca4, Ca5, and Ca6 fluctuation pattern maintained and did not fluctuate more than 21.4 Å^2^ within the simulation period. In comparison to the AMS8 lipase simulation without Ca1 and Ca3, the value of SASA increased indicating the unfolding of AMS8 lipase within the simulation period. Unfolding of the AMS8 lipase structure in the absence of Ca1 and Ca3 was proportional to the structural changes of the AMS8 lipase secondary structure.

The changes in the geometry coordinate and unfolding of the AMS8 lipase structure without Ca1 to Ca6 after MD simulation at 50 ns in water are shown in Figures S2–S7. The MD simulation snapshots at 0, 10, 20, 30, 40, and 50 ns were analyzed. Based on the analysis, the removal of Ca^2+^ ions bound to the RTX parallel *β*-roll motif repeat structure caused destabilization and unfolding of the AMS8 lipase structure. After the removal of Ca3, Ca4, and Ca5, the AMS8 lipase structure seemed to unfold and be unstable, especially at the non-catalytic domain including the RTX parallel *β*-roll motif repeat structure. These conditions proved the critical role of calcium-binding in the folding and stabilization of the RTX parallel *β*-roll motif repeat and the overall AMS8 lipase structures.

### 2.3. Destabilization of RTX Parallel β-Roll Motif Repeat the Structure of AMS8 Lipase Structure after 50 ns MD Simulations

**Figure 5:**
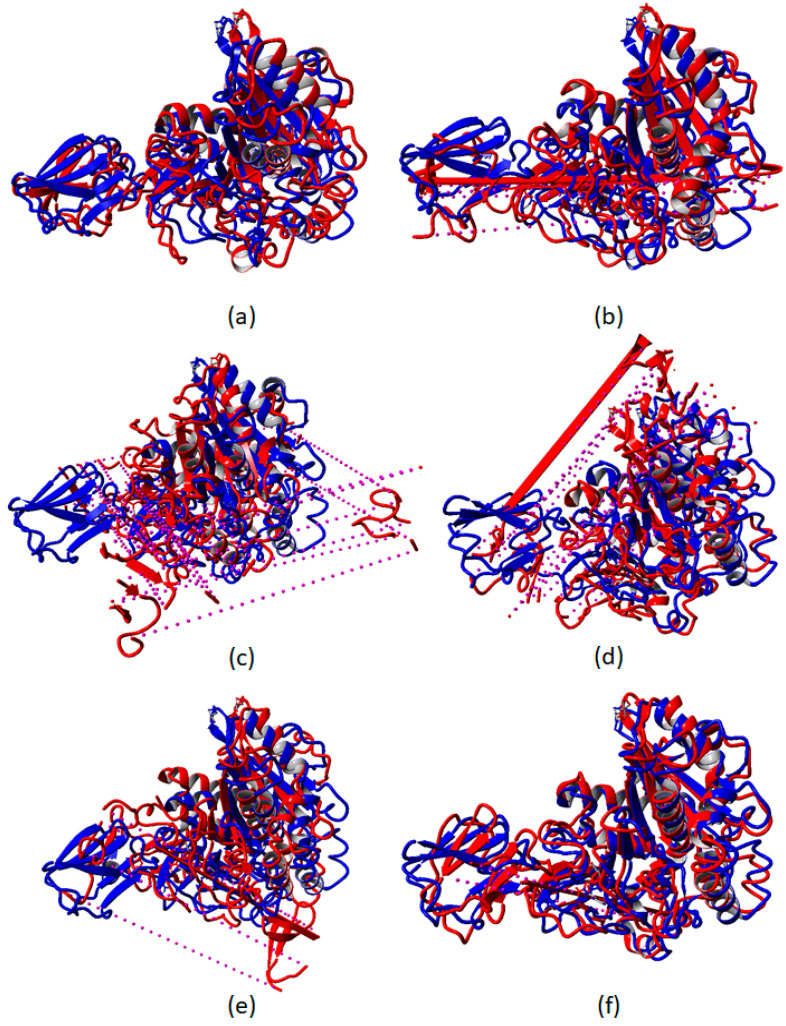
Superposition of the AMS8 lipase structure after MD simulations with the initial structure. (**a**–**f**) represent the superposed images of AMS8 lipase after 50 ns MD simulations in water without Ca1, Ca2, Ca3, Ca4, Ca5, and Ca6, respectively. The blue represents the initial AMS8 lipase structure and the red represents the simulated AMS8 lipase structure. Purple dotted lines represent the domino effect.

After 50 ns MD simulations, the simulated AMS8 lipase structure was superposed with the initial AMS8 lipase structure to determine the superposition value (Å) between the two structures (Figure 5). The superposition values (Å) represent the changes in the geometry coordinate and unfolding of the AMS8 lipase by removal of the specific Ca^2+^ ion from the AMS8 lipase structure after MD simulations. The superposition value (Å) of AMS8 lipase without Ca1, Ca2, Ca3, Ca4, Ca5, and Ca6, individually with the initial structure of AMS8 lipase with all six Ca^2+^ ions are 6.9388, 13.2515, 22.8079, 15.2154, 16.7896, and 7.7949 Å, respectively.

By the removal of Ca3 (22.8 Å), Ca4 (15.2 Å), and Ca5 (16.8 Å), the superposition value was given the highest value. The higher the superposition value indicated the vast differences between the two structures analyzed. All three Ca^2+^ ions (Ca3, Ca4, and Ca5) were bound to the RTX nonapeptide repeat sequence that built the RTX parallel *β*-roll motif repeat structure at the non-catalytic domain of the AMS8 lipase structure. As reported previously, the RTX parallel *β*-roll motif repeat structure was responsible for the stabilization of the whole protein structure. In addition, the presence of Ca^2+^ ions was also relevant for both the folding and stability of many protein domains [5]. By removing the Ca^2+^ ion attached to the RTX parallel *β*-roll motif repeat structure, the whole AMS8 lipase structure, especially the non-catalytic domain became unstable and unfolded at the end of the simulations (Figure 5).

**Figure 6:**
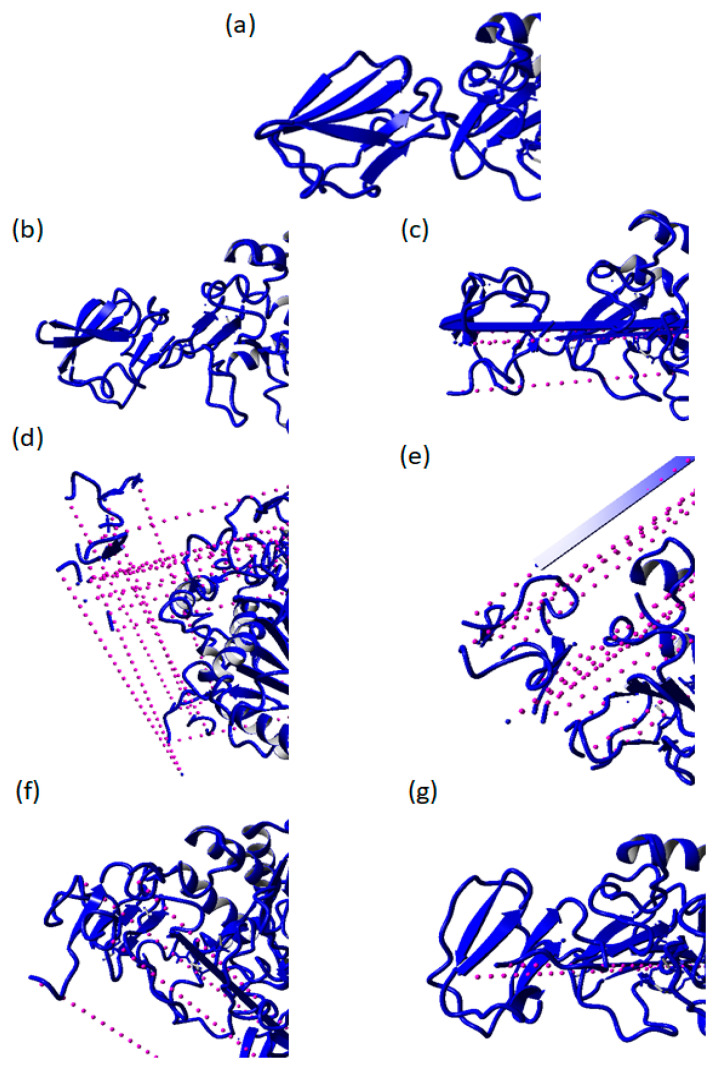
Destabilization of the non-catalytic domain including the release-in-toxin (RTX) parallel *β*-roll motif repeat structure after 50 ns MD simulations. (**a**) Represents the initial AMS8 lipase structure. (**b**–**g**) represent the AMS8 lipase structure without Ca1, Ca2, Ca3, Ca4, Ca5, and Ca6, individually after 50 ns MD simulations. The purple dotted lines represent the domino effect.

In addition, the removal of Ca2, Ca3, Ca4, Ca5, and Ca6 had the domino effect. Parts of the molecules were broken and diffused out of the simulation box at the end of the simulation, as shown by the dotted purple lines in Figure 6. The binding and release of Ca^2+^ ions changed the structural properties of the involved calcium-binding proteins [26]. The formation of *β*-sheet was sustained by calcium-binding. In contrast, in the absence of Ca^2+^ ion, the RTX parallel *β*-roll motif repeat structure appeared to be disordered [27,28]. A previous study showed that the RTX parallel *β*-roll motif repeat structure became disordered and adequately unfolded in the absence of Ca^2+^ ion [6,7,8,27,28,29]. In agreement with our previous laboratory work (CD spectra), in the absence of Ca^2+^ ion (0 mM CaCl_2_), the AMS8 lipase secondary structure, especially *β*-sheet content was decreased [14]. The decrement in *β*-sheet content was due to the disordered RTX domain of the AMS8 lipase in the absence of Ca^2+^ ion.

## 3. Conclusions

The RTX parallel *β*-roll motif repeat structure plays a crucial role in the folding and stabilization of the RTX protein structure. Previous laboratory experiments have been done to study the contribution of Ca^2+^ ion toward the activity and folding of AMS8 lipase. However, laboratory experiments only give data based on static entities. To better understand the influence of Ca^2+^ ion toward the AMS8 lipase functional dynamics and structural conformation changes, we conducted an in silico analysis to complete our funding. The use of bioinformatics approaches: Molecular docking and MD simulations will give additional information on the functional dynamics and structural conformation changes of the AMS8 lipase behavior. The results from molecular docking and MD simulation analysis showed that the removal of Ca^2+^ ions (Ca3, Ca4, and Ca5), especially the removal of Ca3, caused the destabilization and unfolding of the RTX domain in the AMS8 lipase structure. Here, findings from the in silico analysis support the results from the previous laboratory work and provided clear understanding based on the functional dynamics and structural conformation changes of AMS8 lipase that cannot be visualized during the laboratory experiment. The contribution of calcium-binding in the RTX domain proves to be vital for the function as well as sustaining the structural integrity of the enzyme.

## 4. Materials and Methods

### 4.1. AMS8 Lipase Sequence and Predicted Three-Dimensional (3D) Structure

The AMS8 lipase 3D structure was used to perform molecular docking and MD simulation. The three-dimensional (3D) structure (S1) of cold-adapted lipase isolated from the *Pseudomonas* sp. strain AMS8 (GenBank accession no.: ADM87309.1) obtained from Ali et al. [13] was used in this study. The sequence analysis revealed that AMS8 lipase consists of 1431 bp nucleotides that encoded a polypeptide consisting of 476 amino acids [13]. A previous study has adopted the homology modeling approach to predict the AMS8 lipase structure. The templates used for the modeling were the crystal structures of the lipases obtained from *Serratia marcescens* [30] and *Pseudomonas* sp. MIS38 [31]. The atomic coordinates for the lipases *Serratia marcescens* (PDB ID: 2qua) and *Pseudomonas* sp. MIS38 (PDB ID: 2z8x) were obtained from the Protein Data Bank. The 3D model was generated using the YASARA [17]. The validation was performed with VERIFY3D [32] (to evaluate the fitness of the protein sequence in its current 3D structure) and Ramachandran plot [33] (to evaluate the geometrical aspects of the structure). Based on the validated structure done by Ali et al. [13], the structure model of AMS8 lipase was overall good. The model had 89.5% of the residues residing in the most favored allowed region. Although the best scores are 90.0% and higher, the score obtained is considered to be acceptable because the model is a prediction model and not a crystal structure (i.e., the crystal structure is a fully solved structure compared to the predicted one) [13].

### 4.2. Molecular Docking of AMS8 Lipase Structure

The metal ion docking of the AMS8 lipase with Ca^2+^ ion was performed through dock_run.mcr.macro tools from the YASARA software [16]. The AutoDock method follows the Lamarckian Genetic Algorithm and Empirical Binding-free Energy Function. Force field Amber14 (YASARA version 17.4.17) was used to calculate the ligand. The AMS8 metal ion-docking protocol was employed to predict the scoring and binding interactions between the receptor (AMS8 lipase) and the ligand (Ca^2+^ ion). The prepared ligand was docked into the Ca^2+^ ion binding site of the AMS8 lipase. Each ligand selected was docked individually in the targeted binding sites. The docking was subjected to 25 runs, each docking is sorted by the strongest binding energy (kcal/mol). Then, it conducts conformational cluster analysis of the docked conformations to determine which are similar, reporting the clusters ranked by increasing energy. At the end of each docking, AutoDock determined the dissociation constant (k_D_), the coordinates of the docked conformation, and the estimated free energy of binding. The docking results were selected based on the strongest binding energy and the image from the highest binding energy was extracted. LigPlot^+^ was used to generate a 2D representation of ligand-protein interactions from docking images (https://www.ebi.ac.uk/thornton-srv/software/LIGPLOT/). The images were visualized using the Chimera software [34].

### 4.3. Molecular Dynamics (MD) Simulation Parameter of AMS8 Lipase

YASARA software (version 17.4.17) [16] was used for the MD simulation of the AMS8 lipase structure in the presence of different Ca^2+^ ions (Ca1, Ca2, Ca3, Ca4, Ca5, and Ca6). The AMS8 lipase structure in PDB format was loaded and a simulation box (X = 77.49 Å, Y = 74.03 Å, Z = 88.15 Å) was created. Each box was filled with water (0.997 g/mL) at a pressure control of 1 bar. MD simulations involved the simulation of the predicted model inside a trajectory box filled with 6940 molecules of the solvent (including NaCl (0.9%), water, and Ca^2+^ ion) at 25 °C (pH 8.0). Energy minimization was performed using the steepest descent method for about ~2000 steps before the actual simulation began. After minimization, the MD simulations were performed following the achieved density equilibration in a fixed-size simulation box at 90° on every edge. The system was equilibrated at the steepest descent parameters using a time step of 1 fs for intramolecular forces calculating the intramolecular forces in every two simulation sub-steps. The AMS8 lipase structure was energy-minimized with an AMBER03 [35] force field using a cut off 10.486 Å and the Particle Mesh Ewald algorithm [36] to treat long-range electrostatic interactions. The six MD simulations were run up to 50 ns to elucidate the dynamic properties of the enzyme in water in the presence of different Ca^2+^ ions (Ca1, Ca2, Ca3, Ca4, Ca5, and Ca6). Each of the Ca^2+^ ions had been removed in every single simulation, respectively. For example, only Ca1 had been removed in the first simulation. Next, only Ca2 had been removed in the second simulation, and it would continue until the sixth simulation.

### 4.4. Molecular Dynamics (MD) Simulation Analysis of AMS8 Lipase

The AMS8 lipase MD simulation was run up to 50 ns of the production period. The simulation would provide a better understanding of the dynamics properties of the AMS8 lipase structure in water at a different number of Ca^2+^ ions. The root mean square deviation (RMSd) was examined to study the stability of the trajectories. The root mean square fluctuation (RMSf) was computed per residue to explore the flexibility of the trajectories of the protein structure. In both analyses, the greater deviation from the native enzyme structure indicated the disruption of the molecular forces of the molecules. Further analysis was performed by calculating the radius of gyration (Rgyration) and solvent accessible surface area (SASA). Images were visualized using the Chimera software [34]. After 50 ns MD simulations, the simulated AMS8 lipase structure was superposed with the initial AMS8 lipase structure to determine the superposition value between the two structures. The superposition values represented the changes in the geometry coordinate and unfolding of the AMS8 lipase by the removal of Ca^2+^ ion from the structure. The higher the superposition value indicated the vast differences between the two structures analyzed. The initial structure without the removal of any Ca^2+^ ion can be used to compare the structural changes, especially for analyzing the folding/unfolding of the protein structure. In addition, a superposition can also show the domino effect based on changes in RMSD of the two structures analyzed.

## Figures and Tables

**Table 1:** The dissociate constant (pM) and binding energy (kcal/mol) values of metal ion (Ca^2+^) docking analysis of the AMS8 lipase structure.

Ca^2+^ Ion	Dissociate Constant (pM)	Binding Energy [kcal/mol]	Number of Interacting Residues
Ca1	113,732,214,784.0	1.2980	4
Ca2	202,227,302,400.0	0.9470	4
Ca3	160,478,724,096.0	1.0840	5
Ca4	110,701,961,216.0	1.3040	3
Ca5	110,515,273,728.0	1.3050	3
Ca6	185,235,095,552.0	0.9990	2

## Supplementary Materials

The following are available online at https://www.mdpi.com/2072-6651/12/9/579/s1, Figure S1: The 3D structure of AMS8 lipase; Figure S2: Changes in the geometry coordinate and unfolding of the AMS8 lipase without Ca1 after simulation at 50 ns; Figure S3: Changes in the geometry coordinate and unfolding of the AMS8 lipase without Ca2 after simulation at 50 ns; Figure S4: Changes in the geometry coordinate and unfolding of the AMS8 lipase without Ca3 after simulation at 50 ns; Figure S5: Changes in the geometry coordinate and unfolding of the AMS8 lipase without Ca4 after simulation at 50 ns; Figure S6: Changes in the geometry coordinate and unfolding of the AMS8 lipase without Ca5 after simulation at 50 ns; Figure S7: Changes in the geometry coordinate and unfolding of the AMS8 lipase without Ca6 after simulation at 50 ns.
