# Supplementary material for: The Influence of Calcium toward Order/Disorder Conformation of Repeat-in-Toxin (RTX) Structure of Family I.3 Lipase from Pseudomonas fluorescens AMS8
Source: Toxins (Basel). 2020 Sep 9;12(9):579. doi: 10.3390/toxins12090579 (PMC7551394; doi:10.3390/toxins12090579)
Supplement: Supplementary file 1 [file toxins-12-00579-s001.pdf]

Supplementary Materials: The Influence of Calcium toward Order/Disorder Conformation of Repeat-in-Toxin (RTX) Structure of Family I.3 Lipase from *Pseudomonas fluorescens* AMS8

Nur Shidaa Mohd Ali, Abu Bakar Salleh, Thean Chor Leow, Raja Noor Zaliha Raja Abd Rahman and Mohd Shukuri Mohamad Ali

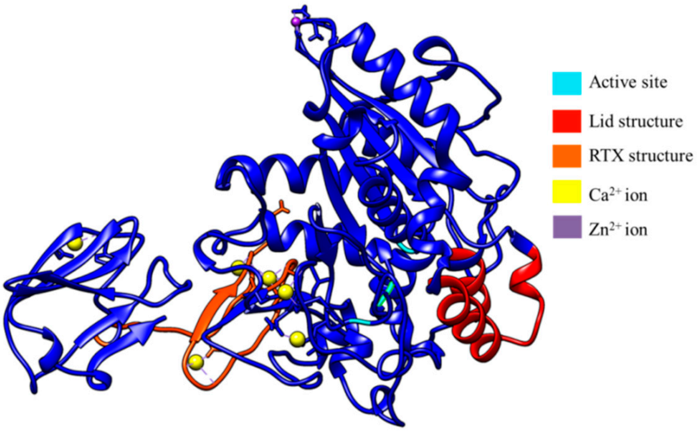

Figure S1. The 3D structure of AMS8 lipase. The AMS8 structure figure was modified from Ali et al. (2013).

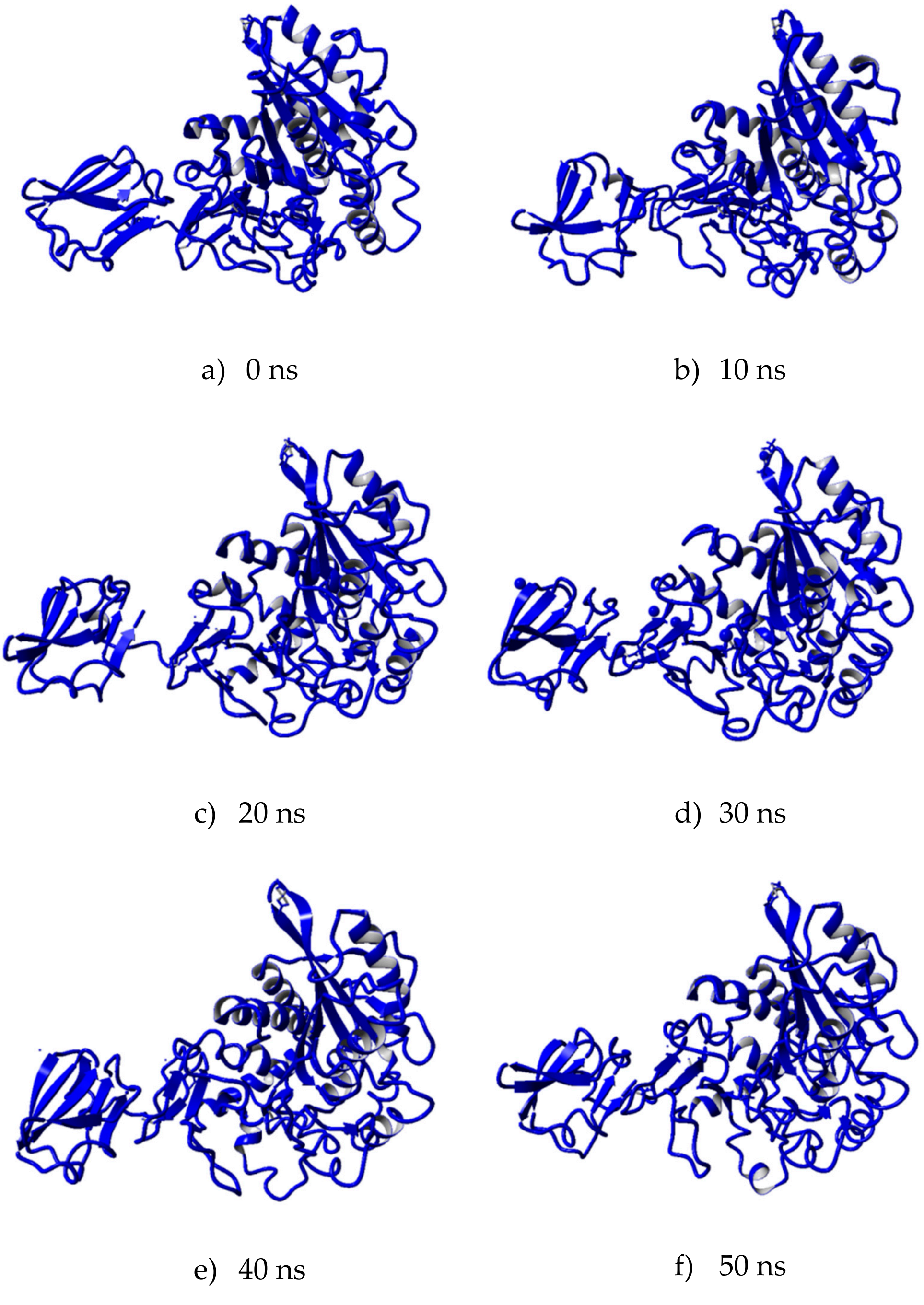

Figure S2. Changes in the geometry coordinate and unfolding of the AMS8 lipase without Ca1 after simulation at 50 ns. (**a**) represent structure of AMS8 lipase before simulation, while (**b**) to (**f**) represent 3D structure of AMS8 lipase after simulated without Ca1 at 10, 20, 30, 40 and 50 ns correspondingly showing for overall structural changes after removal Ca1.

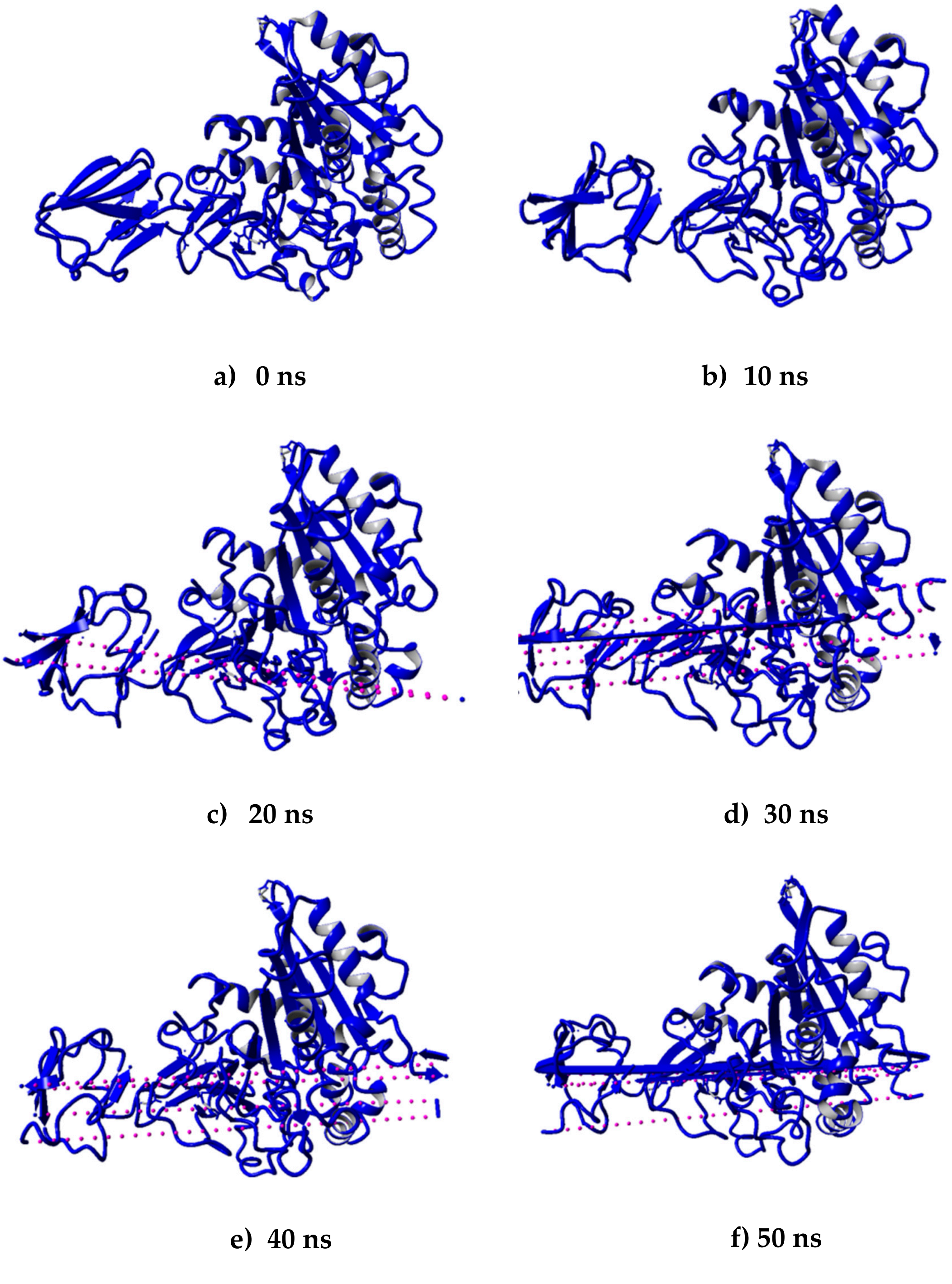

Figure S3. Changes in the geometry coordinate and unfolding of the AMS8 lipase without Ca2 after simulation at 50 ns. (**a**) represent structure of AMS8 lipase before simulation, while (**b**) to (**f**) represent 3D structure of AMS8 lipase after simulated without Ca2 at 10, 20, 30, 40 and 50 ns correspondingly showing for overall structural changes after removal Ca.

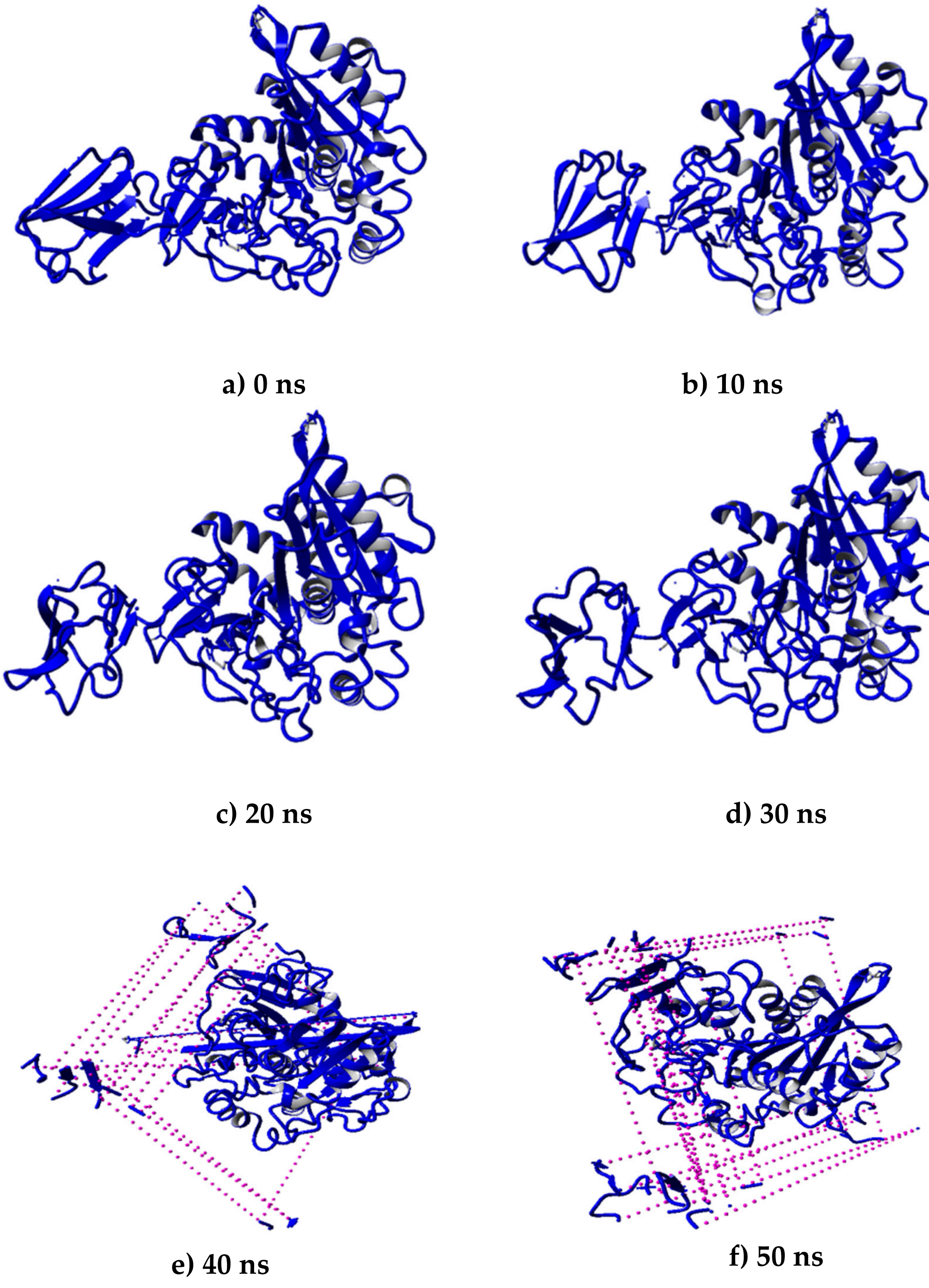

Figure S4. Changes in the geometry coordinate and unfolding of the AMS8 lipase without Ca3 after simulation at 50 ns. (**a**) represent structure of AMS8 lipase before simulation, while (**b**) to (**f**) represent 3D structure of AMS8 lipase after simulated without Ca3 at 10, 20, 30, 40 and 50 ns correspondingly showing for overall structural changes after removal Ca3.

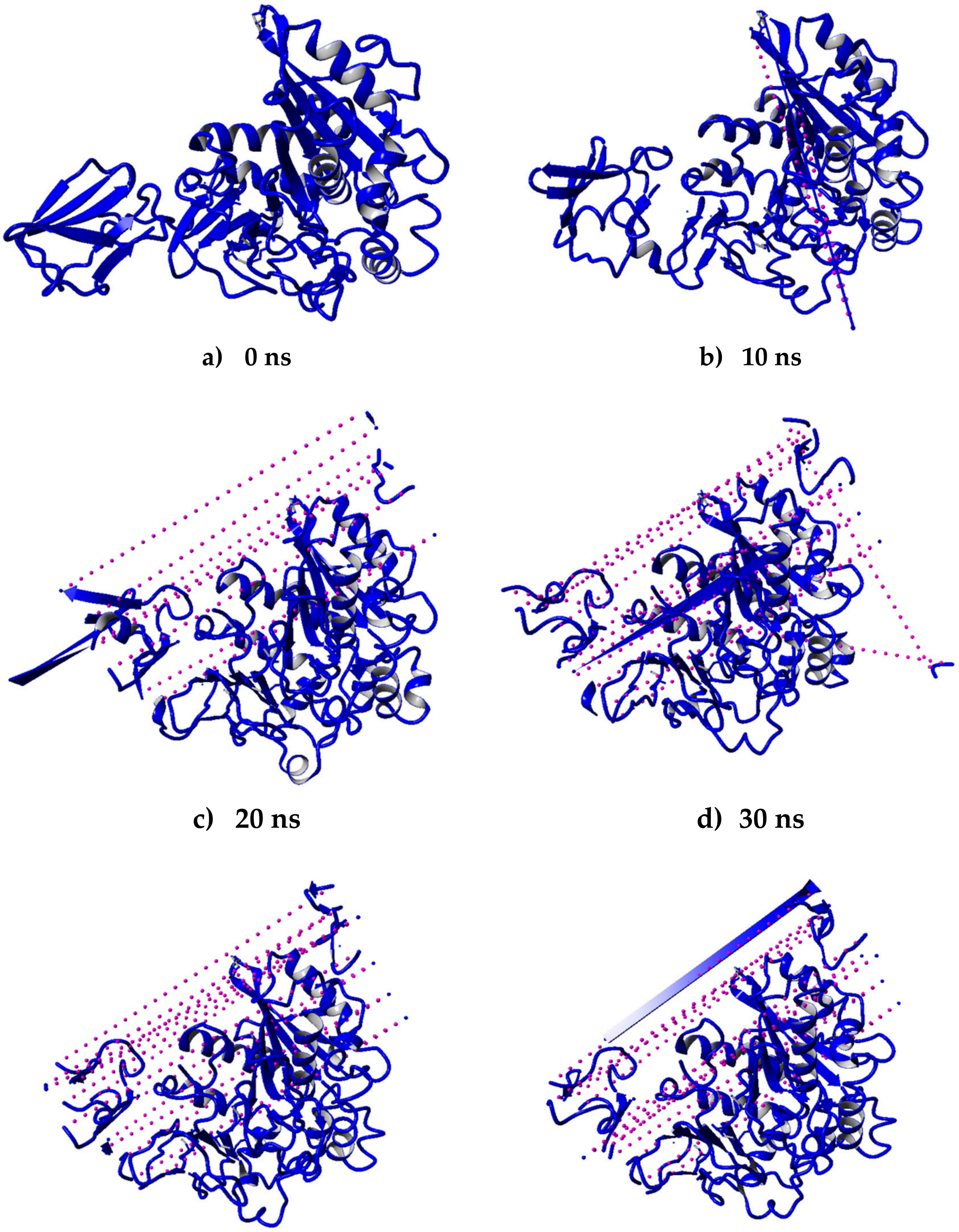

Figure S5. Changes in the geometry coordinate and unfolding of the AMS8 lipase without Ca4 after simulation at 50 ns. (**a**) represent structure of AMS8 lipase before simulation, while (**b**) to (**f**) represent 3D structure of AMS8 lipase after simulated without Ca4 at 10, 20, 30, 40 and 50 ns correspondingly showing for overall structural changes after removal Ca4.

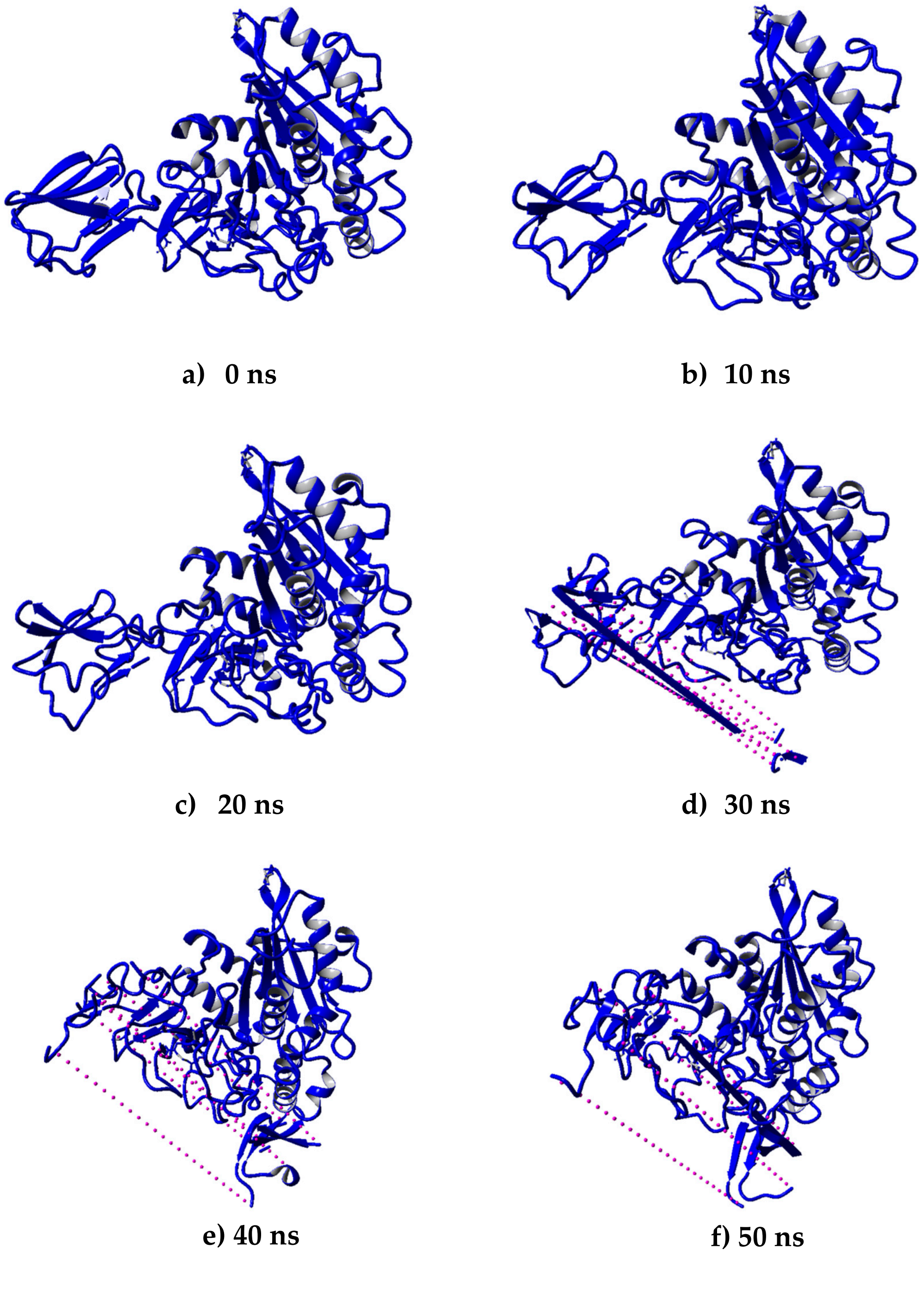

a) 0 ns **b) 10 ns**

c) 20 ns **d) 30 ns**

e) 40 ns **f) 50 ns**

Figure S6. Changes in the geometry coordinate and unfolding of the AMS8 lipase without Ca5 after simulation at 50 ns. (**a**) represent structure of AMS8 lipase before simulation, while (**b**) to (**f**) represent 3D structure of AMS8 lipase after simulated without Ca5 at 10, 20, 30, 40 and 50 ns correspondingly showing for overall structural changes after removal Ca5.

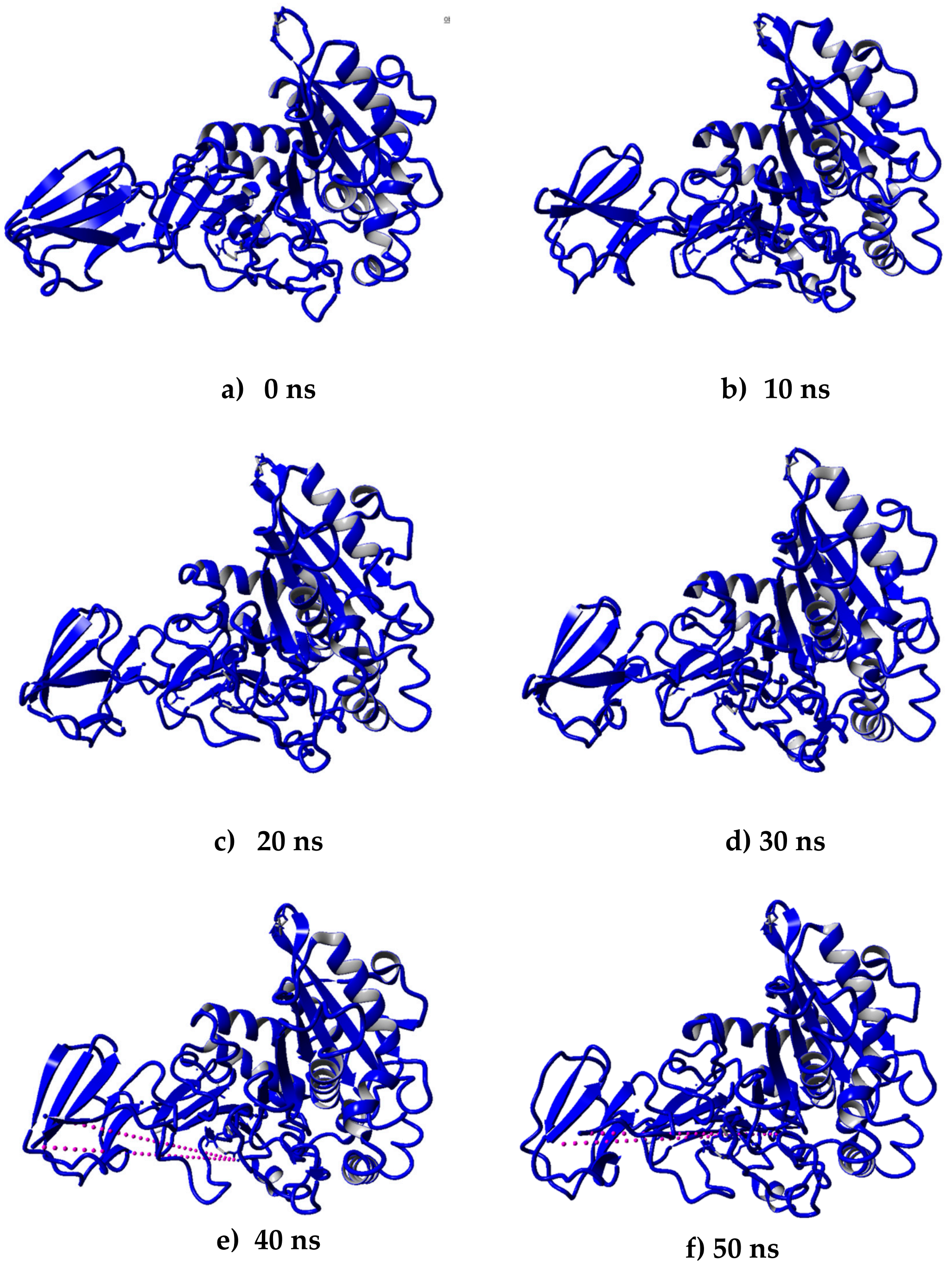

Figure S7. Changes in the geometry coordinate and unfolding of the AMS8 lipase without Ca6 after simulation at 50 ns. (**a**) represent structure of AMS8 lipase before simulation, while (**b**) to (**f**) represent 3D structure of AMS8 lipase after simulated without Ca6 at 10, 20, 30, 40 and 50 ns correspondingly showing for overall structural changes after removal Ca6.
